# Inflammation in areas of fibrosis precedes loss of kidney function in lupus nephritis

**DOI:** 10.1136/lupus-2025-001687

**Published:** 2025-11-28

**Authors:** Silvia Malvica, Paride Fenaroli, Chen-Yu Lee, Sarah Louis, Alessandra Ida Celia, Serena Bagnasco, Xiaoping Yang, Daniel Salvetti, Jeffrey Hodgin, H Michael Belmont, Peter Izmirly, Jill P Buyon, Laurence Magder, Michelle A Petri, The Accelerating Medicines Partnership: RA/SLE Network, Avi Rosenberg, Andrea Fava

**Affiliations:** 1 Department of Pathology, Johns Hopkins University, Baltimore, Maryland, USA; 2 Clinical and Research Laboratory on Urinary Sediment, UOC Nefrologia, Dialisi e Trapianto di Rene, Fondazione IRCCS Ca’ Granda Ospedale Maggiore Policlinico, Milan, Italy; 3 Nephrology and Dialysis Unit, Azienda USL—IRCCS di Reggio Emilia, Reggio Emilia, Italy; 4 Division of Rheumatology, Johns Hopkins University, Baltimore, Maryland, USA; 5 University of Rome La Sapienza, Rome, Italy; 6 Department of Pathology, University of Michigan, Ann Arbor, Michigan, USA; 7 Rheumatology, NYU Grossman School of Medicine, New York City, New York, USA; 8 University of Maryland Medical Center, Baltimore, Maryland, USA

**Keywords:** Lupus Nephritis, Risk Factors, Inflammation

## Abstract

**Background:**

Interstitial fibrosis in lupus nephritis (LN) is often infiltrated by immune cells but typically regarded as non-specific ‘scar reaction’. This study aimed to investigate the relationship between inflammatory fibrosis and kidney disease progression in LN.

**Methods:**

Interstitial fibrosis and tubular atrophy (IFTA) were scored in 124 LN kidney biopsies. Inflammation in areas of IFTA (i-IFTA) was graded 0–3 according to the Banff Classification of Allograft Pathology. Significant glomerular filtration rate (GFR) loss was defined as a decline of >15 mL/min at 3 years from biopsy. Immune cell phenotype was defined by serial immunohistochemistry (13-plex).

**Results:**

IFTA was observed in 88/124 (71%) biopsies, and i-IFTA was identified in 76/88 (86%) cases. The distribution of i-IFTA grades was heterogeneous across all IFTA grades. In patients with moderate-to-severe IFTA (>25%), the degree of i-IFTA was associated with a higher risk of significant GFR loss: 0/1 (0%), 0/3 (%), 3/4 (75%) and 7/9 (78%) for i-IFTA grades 0, 1, 2 and 3, respectively (p=0.015). Multiplexed histology revealed that i-IFTA was mostly composed of CD163+ macrophages and CD4 T cells, followed by CD8 T cells and granulocytes.

**Conclusion:**

I-IFTA is frequently observed in LN and is dominated by macrophages and T cells. For patients with baseline IFTA >25%, the degree of i-IFTA emerged as a predictor of GFR loss. These data support the routine scoring of i-IFTA in LN due to its prognostic implications and nominate i-IFTA as a potential therapeutic target.

**WHAT IS ALREADY KNOWN ON THIS TOPIC**

- Interstitial fibrosis and tubular atrophy (IFTA) is a major determinant of kidney survival in lupus nephritis and other kidney diseases. Inflammation within fibrotic areas (i-IFTA) is often seen on kidney biopsies but has been considered a non-specific ‘scar reaction’, and its prognostic value in lupus nephritis has not been systematically studied.

**WHAT THIS STUDY ADDS**

- We show that i-IFTA is common and heterogeneous in lupus nephritis and that its severity modifies the prognostic impact of IFTA. Among patients with moderate-to-severe IFTA (>25%), higher i-IFTA grades were strongly associated with increased risk of significant glomerular filtration rate loss, while absence of i-IFTA was associated with preserved kidney function.

**HOW THIS STUDY MIGHT AFFECT RESEARCH, PRACTICE OR POLICY**

- These findings support the routine evaluation and reporting of i-IFTA in lupus nephritis biopsies. They also nominate i-IFTA as a potential biomarker and therapeutic target for interventions aimed at slowing fibrosis progression and improving long-term kidney outcomes.

## Introduction

 Lupus nephritis (LN) is a major cause of morbidity and mortality in patients with systemic lupus erythematosus (SLE), leading to end-stage kidney disease (ESKD) in 10%–30% of cases.^1 2^ LN is currently classified into six classes based on the severity and distribution of glomerular histological features.^3^ Kidney histology also provides information on the degree of intrarenal LN activity (inflammation) and chronicity (damage) most commonly quantified by the National Institutes of Health (NIH) Activity Index (AI) and Chronicity Index (CI).^3^ Current understanding of LN’s natural history suggests an evolution from an active, inflammatory phase to a resolving phase, where acute lesions may progress to fibrotic scarring through a process similar to wound healing. Within this framework, histological activity reflects potentially treatable lesions, while chronicity denotes irreversible damage. Chronicity in the diagnostic biopsy, but not activity, is the strongest predictor of future kidney function loss and progression to ESKD in LN.^4^,^9^ The CI is a composite score reflecting the extent of glomerulosclerosis, fibrous crescents, interstitial fibrosis and tubular atrophy.^3^ Interstitial fibrosis and tubular atrophy (IFTA) is the component of CI that most strongly predicts negative renal outcomes in LN^10^,^13^ as well as in other glomerular diseases such as IgA nephropathy^14 15^ and in the kidney allograft.^16^

An inflammatory infiltrate in areas of IFTA (i-IFTA) is often observed, but it is generally regarded as a non-specific feature in native kidney biopsies (scar reaction) rather than active disease requiring treatment. I-IFTA is also observed in non-immunological kidney disease such as diabetic nephropathy and hypertensive kidney disease, with similarly unclear clinical implications. In contrast, in kidney allograft pathology, i-IFTA is regarded as a lesion of immunological significance and is one of the histologic criteria of chronic active cellular-mediated rejection.^17^ However, the evidence supporting immunosuppressive treatment in this context remains inconclusive.^10 18^

The clinical implications of i-IFTA in LN are poorly understood. The objective of this study was to investigate the association between inflammatory fibrosis and kidney disease progression in LN. Additionally, we characterised the immunophenotype of the inflammatory i-IFTA.

## Methods

### Study design and patient population

This study included patients enrolled in the Accelerating Medicines Partnership in rheumatoid arthritis (RA)/SLE cohort as previously described.^19^,^21^ In brief, SLE patients over 18 year of age were enrolled if they fulfilled the revised American College of Rheumatology or the SLICC classification criteria for SLE, underwent a clinically indicated renal biopsy (defined as a urine protein-to-creatinine ratio (UPCR) >0.5 g/g)^1922^,^24^ and received a diagnosis of LN. Patients with a history of kidney transplant or pregnant at the time of biopsy were excluded. Only patients with available high resolution, whole digital images of the kidney biopsy light microscopy slides were included in study reported here. Clinical information, including serologies, was collected at the most recent visit before the biopsy. All patients had a urinalysis at screening and urinary tract infections were clinically excluded by the treating physician before recommending a kidney biopsy.

Participants were treated for LN according to the standard of care determined by their treating physician. Laboratory measurements were carried out in local laboratories with abnormal results defined as per the cut-offs of the laboratory.

The estimated glomerular filtration rate (eGFR) was calculated using the Chronic Kidney Disease Epidemiology Collaboration formula (CKD-EPI).^25^ Significant loss of kidney function was defined as either reaching ESKD (eGFR <15 mL/min/1.73 m²) or a decline of more than 15 mL/min/1.73 m² within 3 years (±3 months). This threshold is considered clinically relevant by guidelines and has been applied in similar studies of early CKD.^26^

### Histological scoring

Renal biopsies were initially evaluated by a board-certified pathologist at the institution where the biopsy took place and assigned histological classes, activity and CIs according to the International Society of Nephrology/Renal Pathology Society Classification.^3 27^ High resolution, whole digital slide images of formalin-fixed paraffin-embedded (FFPE) tissue stained with H&E, periodic acid-Schiff, periodic acid silver methamine stain and Masson trichrome stain were obtained using virtual microscopy (Concentriq, Proscia, Philadelphia, Pennsylvania; Aperio ImageScope V.12.3.2.8013, Leica Biosystems, Wetzlar, Germany). The digital slides were centrally assessed for IFTA and interstitial inflammation including i-IFTA by two operators and reviewed by a senior renal pathologist (SB). IFTA and i-IFTA were graded 0 to 3 based on the Banff Classification of Allograft Pathology (0: <5%, 1+: 6%–25%, 2+: 26%–50%, 3+: >50% and 0: <10%, 1+: 10%–25%, 2+: 26%–50%, 3+:>50%, respectively).^17^ Scoring of interstitial inflammation was performed avoiding inflammatory infiltrates in areas immediately surrounding sclerosed glomeruli and collection of extravasated Tamm Horsfall protein. This is the first study applying Banff i-IFTA scoring to LN, and no prior validations exist in this specific context. The Banff criteria have been used extensively in transplant pathology, where interstitial inflammation in areas of fibrosis is considered clinically significant.

### Statistical analysis

Descriptive statistics are presented as mean and SD or median and IQR for continuous variables, and frequencies for categorical variables. Analysis of variance (ANOVA), two-tailed Student’s t-test or Wilcoxon’s test were used to compare continuous variables, and Fisher’s exact test was used to compare categorical variables where appropriate. χ^2^ for trend was used to associate inflammation in interstitial fibrosis and renal outcomes. Trends were tested by the Cochran-Armitage trend test and, in case of small sample size, confirmed by an exact test by permutation.^28^

### Serial immunohistochemistry

Archival FFPE was retrieved for serial immunohistochemical staining. In brief, serial immunohistochemistry (sIHC) involves multiple cycles of staining, destaining and restaining with high-resolution images obtained with each cycle. Tissue sections (5 µm) were deparaffinised and subjected to antigen retrieval using citrate. Slides were blocked for peroxidases and incubated with primary antibodies, secondary horseradish peroxidase (HRP) reagents, 3-amino-9-ethylcarbazole (AEC)-Red Chromogen and Haematoxylin. Slides were decoloured in 90% ethanol, stripped using antibody elution buffer and sequentially cycled through the marker panel (CD3, CD20, CD138, PR3, CD4, CD8, CD14, CD16, CD163, CD66b, CD15, myeloperoxidase (MPO), pankeratin). Images were captured at 40X magnification in each round.

Image files were imported into HALO-AI (V.3.5, Indica Labs) for deconvolution, registration, fusion and cell segmentation to create a registered composite image including all markers at single-cell level. Tissue regions including glomeruli and tubulointerstitium were initially identified by a DenseNet V.2 AI classifier trained on a coregistered periodic acid–Schiff (PAS) and manually validated. Areas of IFTA were manually annotated (SM) and validated by an experienced renal pathologist (SB). Representative multichannel sIHC images were generated using pseudocolours.

Single-cell analysis was performed using R software (V.4.4.1, The R Foundation). Marker intensity was normalised using the centred-log ratio transformation followed by scaling.^29^ Batch correction was performed using Harmony.^30^ The Seurat package (V.5.0.3) was used to perform dimensional reduction with PCA, followed by the construction of a k-nearest neighbour (KNN) graph, and subsequent clustering using the Louvain algorithm.

Cell clusters exhibiting high pankeratin intensity were excluded to filter out renal epithelial cells. Clusters with immune marker levels exceeding 2 SD above the mean were identified as candidate immune cell clusters for density analysis. The paired t-test was used to discover the significantly enriched immune cell types in IFTA or non-IFTA.

### Data attribution

The results published here are in whole or in part based on data obtained from the ARK Portal (arkportal.synapse.org).

## Results

### i-IFTA is a frequently observed feature of LN

We included 124 kidney biopsies from patients with LN who underwent a clinically indicated kidney biopsy and had a UPCR>0.5 g/g. The clinical and demographic features are summarised in table 1. Most patients were female (79.8%), with 50% identifying as black, 25% as white, 11% as Asian and 28.2% as Hispanic. At the time of biopsy, the majority had preserved kidney function and subnephrotic range proteinuria (mean eGFR 82.8 mL/min/1.73m^2^). Proliferative LN (pure class III, IV or mixed with V) was present in 58% of patients, and NIH AI and CI scores varied widely (median AI 3 (range 3–18) and median CI 3 (range 0–9)).

**Table 1:** Clinicodemographic characteristics at the time of kidney biopsy

	Overall	i-IFTA score				P value
		0	1	2	3	
n	124	48	19	25	32	
Age, years (mean (SD))	36.4 (12.7)	36.9 (13.3)	37.9 (15.3)	32.40 (11.1)	39.09 (11.8)	0.442
Female (%)	99 (79.8)	36 (75.0)	16 (84.2)	21 (84.0)	26 (81.2)	0.745
Race (%)						
Asian	14 (11.3)	7 (14.6)	4 (21.1)	1 (4.0)	2 (6.2)	0.17
Black	62 (50.0)	22 (45.8)	7 (36.8)	13 (52.0)	20 (62.5)	0.13
White	31 (25.0)	14 (29.2)	4 (21.1)	7 (28.0)	6 (18.8)	0.38
Other	17 (13.7)	5 (10.4)	4 (21.1)	4 (16.0)	4 (12.5)	0.77
Hispanic (%)	35 (28.2)	8 (16.7)	7 (36.8)	12 (48.0)	8 (25.0)	0.03
eGFR (mean (SD))	82.82 (28.82)	93.35 (22.27)	93.41 (30.49)	78.74 (35.72)	63.46 (22.74)	0.005
UPCR (mean (SD))	2.07 (2.46)	1.20 (0.95)	1.46 (1.43)	2.97 (3.32)	2.91 (3.16)	0.075
First biopsy (%)	49 (40.8)	23 (96)	6 (33)	9 (36)	11 (37)	0.54
ISN class (%)						
I/II	9 (7.3)	5 (10.4)	0 (0.0)	1 (4.0)	3 (9.4)	0.78
Membranous	42 (33.9)	15 (31.2)	8 (42.1)	6 (24.0)	13 (40.6)	0.63
Mixed	35 (28.2)	11 (22.9)	6 (31.6)	10 (40.0)	8 (25.0)	0.58
Proliferative	37 (29.8)	17 (35.4)	5 (26.3)	8 (32.0)	7 (21.9)	0.25
VI	1 (0.8)	0 (0.0)	0 (0.0)	0 (0.0)	1 (3.1)	0.17
IFTA % (mean (SD))	16.50 (19.68)	4.93 (11.40)	15.57 (16.97)	22.55 (19.63)	28.31 (21.69)	<0.001
IFTA grade (%)						
0	36 (29.0)	36 (75.0)	0 (0.0)	0 (0.0)	0 (0.0)	<0.001
1	58 (46.8)	10 (20.8)	14 (73.7)	18 (72.0)	16 (50.0)	0.02
2	21 (16.9)	1 (2.1)	3 (15.8)	5 (20.0)	12 (37.5)	<0.001
3	9 (7.3)	1 (2.1)	2 (10.5)	2 (8.0)	4 (12.5)	0.09
Activity Index (median (range))	3.00 (0–18)	2 (0–18)	3 (0–16)	4 (0–15)	3 (0–15)	0.3
Chronicity Index (median (range))	3 (0–9)	1 (0–6)	3 (0–7)	3.5 (0–9)	5 (1–9)	<0.001
Treatment						
Hydroxychloroquine (%)	106 (88.3)	41 (87.2)	16 (88.9)	24 (96.0)	25 (83.3)	0.89
Mycophenolate (%)	61 (50.8)	28 (59.6)	6 (33.3)	10 (40.0)	17 (56.7)	0.62
Cyclophosphamide (%)	1 (0.8)	0 (0.0)	1 (5.6)	0 (0.0)	0 (0.0)	0.8
Azathioprine (%)	9 (7.5)	5 (10.6)	2 (11.1)	1 (4.0)	1 (3.3)	0.17
Belimumab (%)	2 (1.7)	0 (0.0)	0 (0.0)	1 (4.0)	1 (3.3)	0.17
Tacrolimus (%)	9 (7.5)	4 (8.5)	1 (5.6)	1 (4.0)	3 (10.0)	0.97
ACEi/ARBs (%)	58 (48.3)	22 (46.8)	6 (33.3)	13 (52.0)	17 (56.7)	0.32
Prednisone equivalent dose, mg (median (range))	27.5 (1.25–1250)	30 (1.25–1250)	20 (1.25–1250)	40 (5–1250)	10 (2.5–625)	0.51

Kruskal-Wallis or ANOVA tests were applied based on the distribution of each variable. Test for trends was performed using the Cochrane-Armitage test.

ACEi, angiotensin-converting enzyme inhibitor; ANOVA, analysis of variance; ARBs, angiotensin II receptor blockers; eGFR, estimated glomerular filtration rate; ESKD, end-stage kidney disease; i-IFTA, inflammation in areas of interstitial fibrosis and tubular atrophy; ISN, International Society of Nephrology; UPCR, urine protein-to-creatinine ratio.

**Figure 1:**
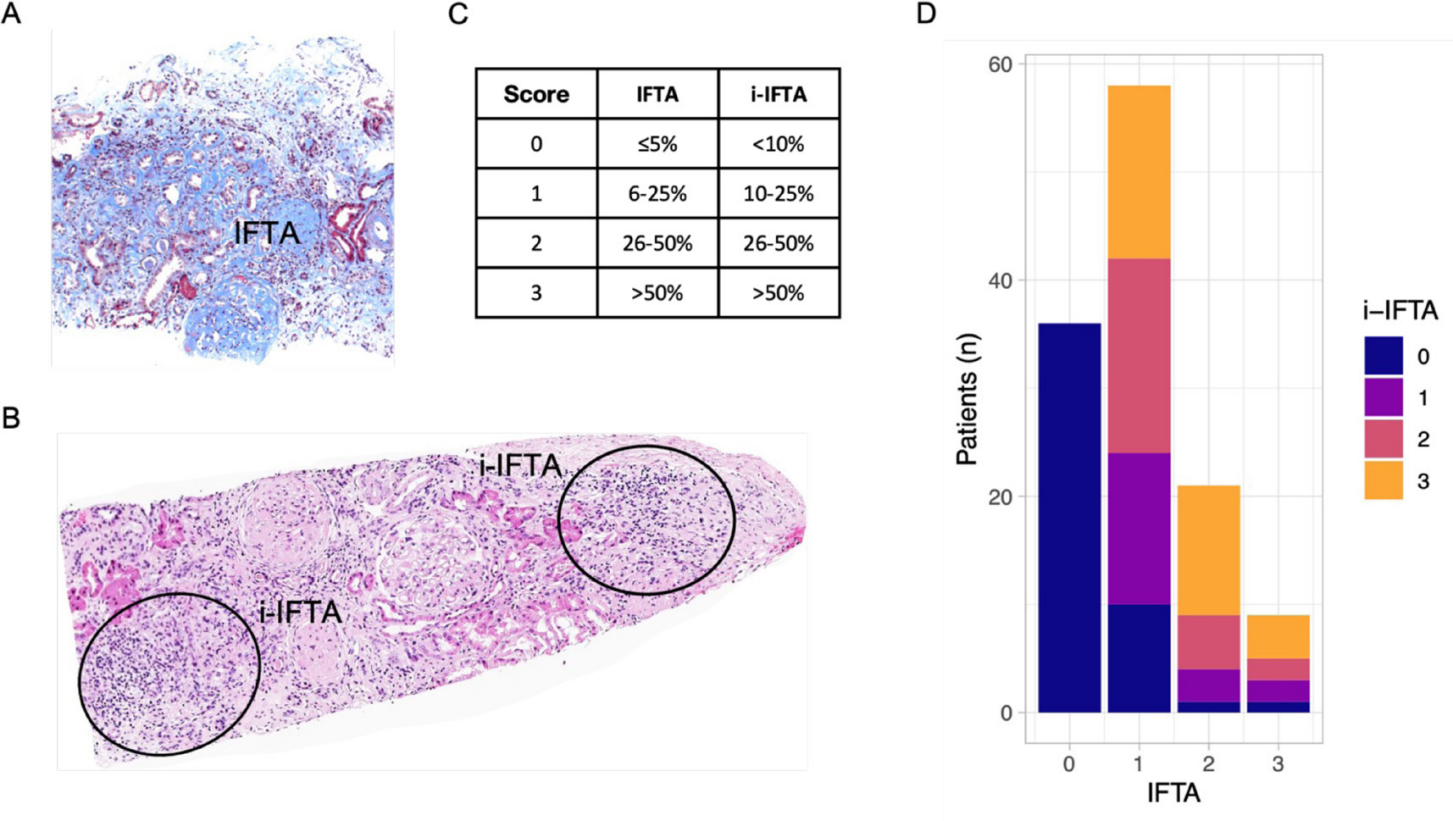
Inflammation in areas of interstitial fibrosis and tubular atrophy (i-IFTA) is frequently observed in lupus nephritis (LN). (**A**) Representative kidney biopsy showing extensive interstitial fibrosis (light blue stain by Trichrome) with interspersed tubular atrophy (iFTA). (**B**) Representative histological image showing an inflammatory infiltrated in areas of IFTA (i-IFTA). (**C**) Grading of IFTA and i-IFTA according to the percentage of fibrotic cortex for IFTA and the percentage of fibrotic cortex with inflammatory infiltrate for i-IFTA as per Banff criteria.^12^ (**D**) Distribution of i-IFTA grades (0–3) according to IFTA grades in 124 LN biopsies showing high frequency and heterogeneity in the degree of i-IFTA in patients with IFTA.

IFTA was observed in 88 of the 124 biopsies (71%), primarily mild: 58 patients (47%) had an IFTA score of 1, 21 patients (17%) had a score of 2 and 9 patients (7%) had a score of 3. I-IFTA was present in 76 of the 88 patients with IFTA (86%), and varying degrees of i-IFTA were distributed similarly across all IFTA score groups >0 (figure 1). Specifically, among the patients with IFTA, 12 (14%) had an i-IFTA score of 0 (<10% of IFTA), 19 (22%) had an i-IFTA score of 1 (10%–25%), 25 (28%) had an i-IFTA score of 2 (25%–50%) and 32 (36%) had an i-IFTA score of 3 (>50%).

No significant associations were found between i-IFTA and age, sex, race or treatment at time of biopsy (table 1). Higher degrees of i-IFTA were associated with worse eGFR (p=0.005) and, numerically, with higher proteinuria (p=0.075). The distribution of i-IFTA was similar across most LN histological classes. There was no association between i-IFTA and the NIH AI, which primarily focuses on glomerular inflammation. However, i-IFTA was significantly associated with higher NIH CI scores (p<0.001).

### i-IFTA precedes loss of kidney function

Next, we tested whether i-IFTA was associated with future loss of kidney function. Longitudinal GFR data at 3 years from the index kidney biopsy were available for 67 patients (table 2). Of these, 26/67 (39%) lost >15 mL/min of GFR or developed ESKD.

**Table 2:** Clinicodemographic data and biopsy results of patients with the 3-year postbiopsy follow-up according to GFR loss status

	GFR loss>15 mL/min or ESKD by 3 years		P value
	No	Yes	
n	41	26	
Age, years (mean (SD))	37.07 (13.28)	36.90 (12.88)	0.962
Female (%)	31 (75.6)	22 (84.6)	0.565
Race (%)			0.649
White	15 (37.5)	8 (30.8)	
Black	17 (42.5)	15 (57.7)	
Asian	3 (7.5)	1 (3.8)	
Other	5 (12.5)	2 (7.7)	
Hispanic (%)	14 (34.1)	7 (26.9)	0.726
eGFR (mean (SD))	83.36 (26.14)	80.54 (28.26)	0.720
UPCR (mean (SD))	1.88 (2.68)	1.97 (1.76)	0.897
First biopsy (%)	23 (56.1)	20 (76.9)	0.141
ISN class (%)			0.05
Membranous	13 (31.7)	16 (61.5)	
Mixed	11 (26.8)	3 (11.5)	
Proliferative	17 (41.5)	7 (26.9)	
Activity (median (range))	2 [0–15)	4 [0–18)	0.202
Chronicity (mean (range))	2 [0–7)	3 [0–9)	0.274
IFTA % (mean (SD))	10 (13.7)	26.3 (23.9)	0.002
IFTA grade (%)			0.142
0	16 (39.0)	5 (19.2)	
1	18 (43.9)	11 (42.3)	
2	6 (14.6)	7 (26.9)	
3	1 (2.4)	3 (11.5)	
i-IFTA grade			0.042
0	19 (46.3)	7 (26.9)	
1	9 (22.0)	2 (7.7)	
2	8 (19.5)	8 (30.8)	
3	5 (12.2)	9 (34.6)	

Kruskal-Wallis or ANOVA tests were applied based on the distribution of each variable.

ANOVA, analysis of variance; eGFR, estimated glomerular filtration rate; ESKD, end-stage kidney disease; i-IFTA, inflammation in areas of interstitial fibrosis and tubular atrophy; ISN, International Society of Nephrology; UPCR, urine protein-to-creatinine ratio.

**Figure 2:**
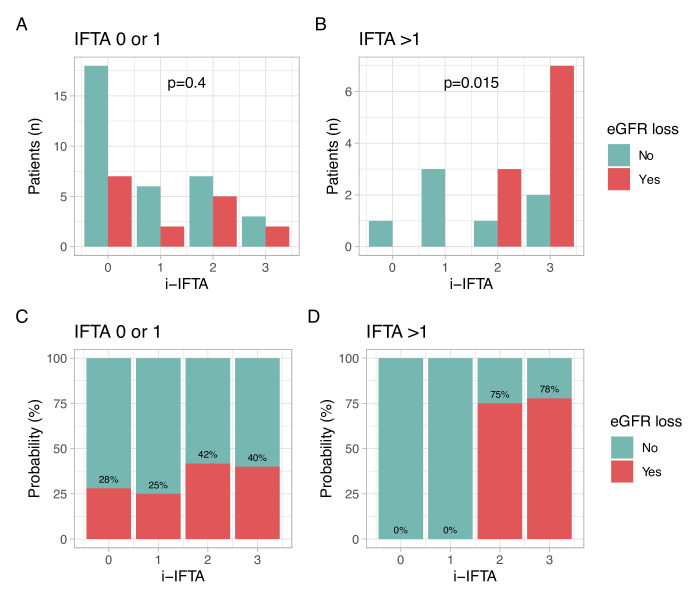
Risk of future GFR loss according to i-IFTA. Distribution of patients who developed significant loss of GFR (>15 mL or ESKD) within 3 years of lupus nephritis (LN) biopsy according to i-IFTA in patients with mild (**A**) or moderate-severe (**B**) IFTA. The results in (A and B) are reported as percentages in (C and D). P for trend was confirmed by an exact test by permutation (A, p=ns; B, p=0.0179).^26^ eGFR, estimated glomerular filtration rate; ESKD, end-stage kidney disease; i-IFTA, inflammation in areas of interstitial fibrosis and tubular atrophy.

As expected, GFR loss was associated with the NIH CI and higher IFTA (table 2). Patients who developed GFR loss had higher i-IFTA scores at baseline. I-IFTA is graded according to the percentage of inflammatory infiltrates within areas of IFTA. Therefore, the total abundance of i-IFTA in the biopsy depends on the underlying amount of IFTA. For example, a mild degree of IFTA such as 10% (grade 1) could be infiltrated in 50% of its area (i-IFTA 3), but the total cortical area of i-IFTA would equal 5%. In contrast, a severe degree of IFTA such as 50% (grade 3) could be infiltrated in 10% of its area (i-IFTA 1), thereby representing the same 5% of total cortical area of i-IFTA. To best analyse the clinical impact of i-IFTA and avoid this potential source of bias, we stratified patients according to the degree of IFTA into two groups: IFTA 0–1 and 2–3 (none to mild, and moderate to severe, respectively). The distribution of i-IFTA grades according to the degree of IFTA is illustrated in figure 2. In patients with none to mild IFTA (grade 0–1, ≤25%), the degree of i-IFTA was not associated with risk of GFR loss. In contrast, in patients with moderate to severe IFTA (grade 2–3, >25%), the degree of i-IFTA was associated with higher risk of eGFR loss (p for trend 0.015). In patients with IFTA 2 or 3, absence of i-IFTA was associated with no increased risk of GFR loss. Conversely, the risk increased with higher degrees of i-IFTA as 0%, 0%, 75% and 78% for i-IFTA 0, 1, 2 and 3, respectively.

### Phenotype of the immune infiltrate in i-IFTA

**Figure 3:**
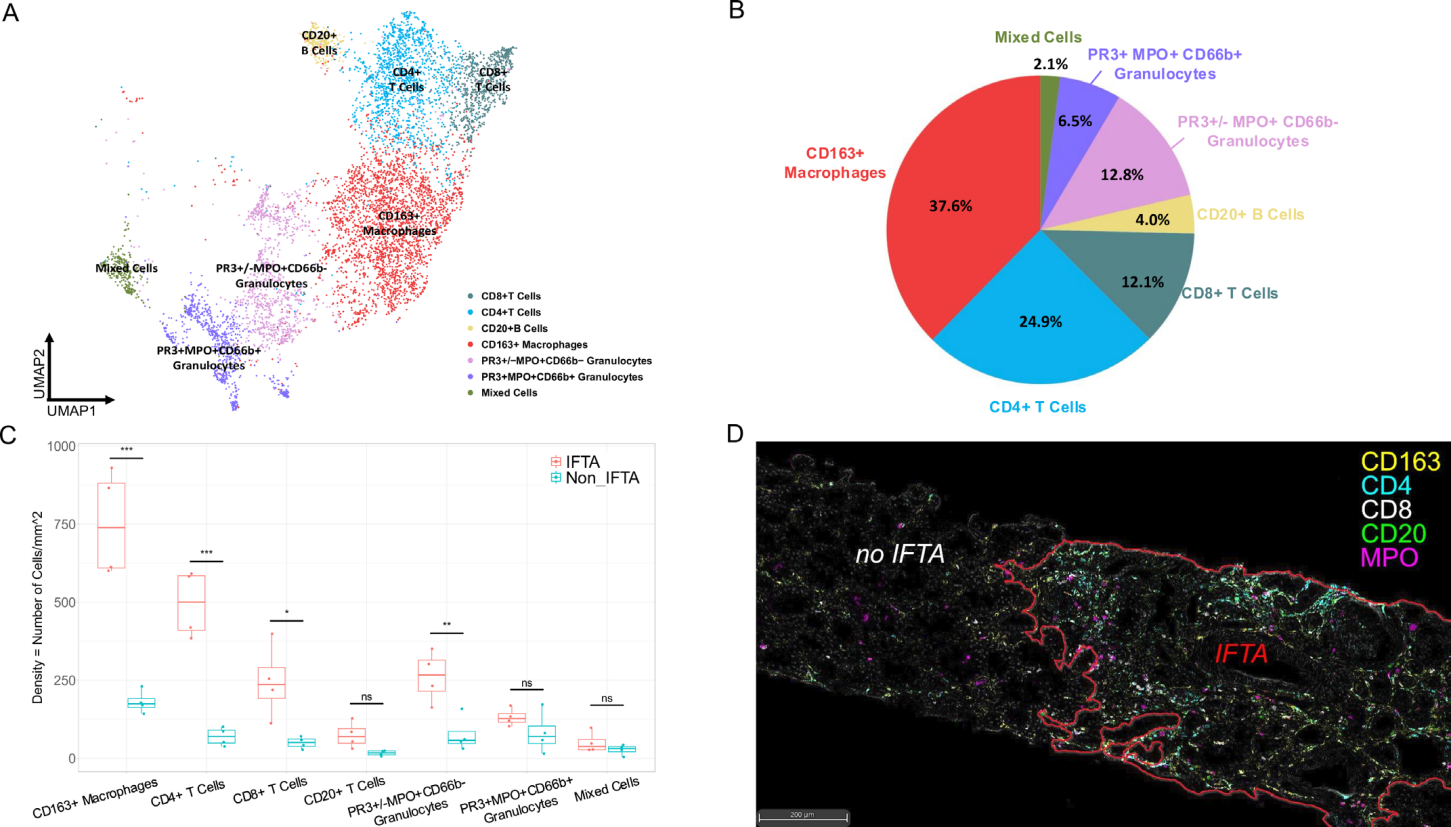
Immune cell composition of i-IFTA. Using 13-plex serial IHC, seven immune cell clusters were identified and annotated based on their marker expression. (**A**) UMAP of 8875 kidney-infiltrating immune cells (including all kidney regions) from four LN biopsies (2 ISN Class III, 1 pure V and 1 VI). (**B**) Pie chart showing the distribution of the immune cell infiltrate in i-IFTA. (**C**) Density of immune cell populations infiltrating areas of IFTA and tubulointerstitial regions without IFTA in four LN kidney biopsies. (**D**) Representative multichannel image showing the difference in immune infiltrate density in IFTA. Pseudocolours from five selected markers are shown. Significance: *p=0.05; **p<0.05; ***p<0.01. IHC, immunohistochemistry; i-IFTA, inflammation in areas of interstitial fibrosis and tubular atrophy; LN, lupus nephritis; MPO, myeloperoxidase; ns, not significant; UMAP, Uniform Manifold Approximation and Projection.

We employed sIHC to characterise the phenotype of the immune infiltrate in i-IFTA. We obtained 47 022 cells from four biopsies, including 8875 immune cells, which clustered into seven groups (figure 3A). These included CD4+T cells, CD8+T cells, CD20+B cells, CD163+macrophages and two clusters of granulocytes. Granulocytes were characterised by MPO expression with one cluster coexpressing PR3 and CD66b suggesting neutrophil lineage whereas the other showed variable PR3 expression but no CD66b suggesting a monocyte lineage (or atypical/non-polylobate neutrophils based on morphology).

The infiltrate in i-IFTA was heterogeneous and included all cell types (figure 3B). CD163+macrophages (37.6%) and CD4 T cells (24.9%) represented the majority of the infiltrate in i-IFTA, followed by CD8 T cells, granulocytes and B cells. Plasma cells were also present in i-IFTA, but these were very rare, with a total of 33 identified in the four biopsies analysed (online supplemental figure 1).

Figure 3C displays the density of each immune cluster in i-IFTA as compared with the infiltrate in tubulointerstitial regions without fibrosis. I-IFTA was characterised by a higher density of most immune cells, especially CD163+macrophages and CD4+T cells. A representative multichannel sIHC image illustrating the increase in CD163+macrophages, CD4+T cells, CD8+T cells and CD66b- granulocytes in IFTA is shown in figure 3D.

## Discussion

In this study, we demonstrated that i-IFTA is frequently observed in LN and showed substantial heterogeneity in its severity. I-IFTA is characterised by a heterogeneous immune infiltrate, primarily composed of CD163+ macrophages and T lymphocytes. Although considered a non-specific finding, the degree of i-IFTA emerged as a predictor of GFR loss, particularly for patients with baseline IFTA>25%.

IFTA is a well-established marker of injury severity and is a strong predictor of CKD progression in various kidney diseases, including in LN.^10^,^13^ Consistent with the previous findings,^10^ our study confirmed that higher IFTA correlates with higher risk of GFR loss. The risk within IFTA was significantly influenced by the degree of the inflammatory infiltrate (i-IFTA). Higher i-IFTA predicted CKD progression in patients with moderate to severe IFTA, while the absence of i-IFTA despite IFTA was associated with no GFR loss. The limited number of patients in the last subgroup limits definitive conclusions. Nonetheless, these findings highlight the prognostic value of i-IFTA and support its routine inclusion and quantification in LN biopsy reports. As expected, IFTA >1 was observed exclusively in patients undergoing repeat biopsies, suggesting that i-IFTA may reflect ongoing injury and contribute to CKD progression in patients with refractory or relapsing LN. The significance of i-IFTA in LN is further reinforced by previous studies, showing that total tubulointerstitial inflammation, rather than inflammation in non-fibrotic regions, is linked to worse renal outcomes.^10 31^ Together, these observations address a critical gap identified in the 2018 International Society of Nephrology (ISN)/Renal Pathology Society (RPS) revision to the histological classification of LN^3^ and should inform the development of future scoring systems.

The association between i-IFTA and GFR loss reinforces the link between inflammation and fibrosis. This is further supported by the critical role of i-IFTA in the diagnosis of chronic active T-cell-mediated rejection^17^ and association with poor kidney survival.^32^,^34^ Similar to other organs, fibrogenesis in the kidneys follows a common pattern where acute injury activates mesenchymal cells, fibroblasts and pericytes, leading to a proinflammatory cascade.^35 36^ This environment triggers myofibroblast proliferation, which promotes collagen secretion and extracellular matrix (ECM) deposition. Under normal conditions, this process is self-limited, with myofibroblasts undergoing apoptosis once the repair process is complete. However, if inflammation persists or injury is repeated, the repair process becomes unbalanced, resulting in excessive ECM deposition, parenchymal distortion and functional decline.^37^ In the kidneys, this leads to scarring, nephron loss and reduced renal function, with key contributors including myofibroblasts, macrophages, transforming growth factor-beta (TGF-β) signalling, epithelial to mesenchymal transition and hypoxia.^38^ Given the association of i-IFTA with GFR loss, we hypothesise that i-IFTA contributes to fibrosis, possibly as a marker of an ongoing inflammatory process^13 39^ that could be targeted therapeutically to improve kidney outcomes. Animal models support this hypothesis, showing that the removal of macrophages after injury can prevent fibrosis.^40^ Previous repeat biopsy studies showed that kidney fibrosis in LN is partially reversible.^41^ Further studies should explore whether targeting i-IFTA can mitigate CKD progression in LN.

The immune infiltrate within i-IFTA was predominantly composed of CD163+ macrophages and CD4+T cells, similar to the patterns observed in other fibrotic diseases and renal disorders such as IgA nephropathy and transplant rejection.^3442^,^44^ In IgA nephropathy, CD68+ macrophage accumulation and CD3+/CD8+T lymphocyte infiltration were linked to disease progression.^44 45^ The association of CD163+ macrophages with decreased renal function is consistent across IgA nephropathy^46^ and kidney transplantation,^47^ suggesting a shared mechanism of damage progression. CD163+ macrophages, which include alternatively activated (M2) pro-resolving and profibrotic phenotypes, are thought to promote fibrogenesis by recruiting and activating myofibroblasts.^48^ Additionally, M2 macrophages may directly contribute to fibrosis through macrophage-myofibroblast transition.^49^ Mechanistic studies demonstrate that M2 macrophages drive the transition from acute injury to chronic kidney disease and that their depletion reduces collagen deposition.^48 49^ The implication of macrophages the fibrosing process is observed across tissues such as in idiopathic pulmonary fibrosis,^23^ liver fibrosis^50^ and cardiac neonatal lupus.^51^ In addition to macrophages, the presence of CD4+ and CD8+ T cells and B cells along with granulocytes and monocytes implicates a broad interaction between the adaptative and innate immune system.^52^ Whether the response within IFTA is driven by a specific antigen as previously observed in interstitial inflammation^53^ remains to be determined.

We acknowledge several limitations of this study. The sample size restricted our ability to conduct subgroup analyses, such as stratifying by ISN class or assessing the impact of medication use. Furthermore, we could not determine the ‘induction’ regimen for each patient. Important confounding factors such as hypertension control, medication adherence or nephrotoxic events could not be fully accounted for. Additionally, key cell types involved in fibrosis, such as fibroblasts and dendritic cells, were not quantified, limiting our ability to study cell–cell interactions systematically. Multiplexed histology was conducted only on four patients, limiting the generalisability of the findings. Larger studies will be necessary to confirm these findings and assess their broader applicability.

In conclusion, our findings establish i-IFTA as a negative prognostic marker in LN, predicting poor renal outcomes. The presence and severity of i-IFTA should be routinely reported in biopsy assessments due to its prognostic significance. Importantly, i-IFTA represents a potential therapeutic target, and future research should focus on whether interventions aimed at reducing interstitial inflammation can slow or prevent CKD progression in LMN and other fibrotic renal diseases.

## Supplementary material

10.1136/lupus-2025-001687 online supplemental figure 1

## Data Availability

Data are available upon reasonable request.
