# Supplementary figures and images for: Inflammation in areas of fibrosis precedes loss of kidney function in lupus nephritis
Source: Lupus Sci Med. 2025 Nov 28;12(2):e001687. doi: 10.1136/lupus-2025-001687 (PMC12666189; doi:10.1136/lupus-2025-001687)

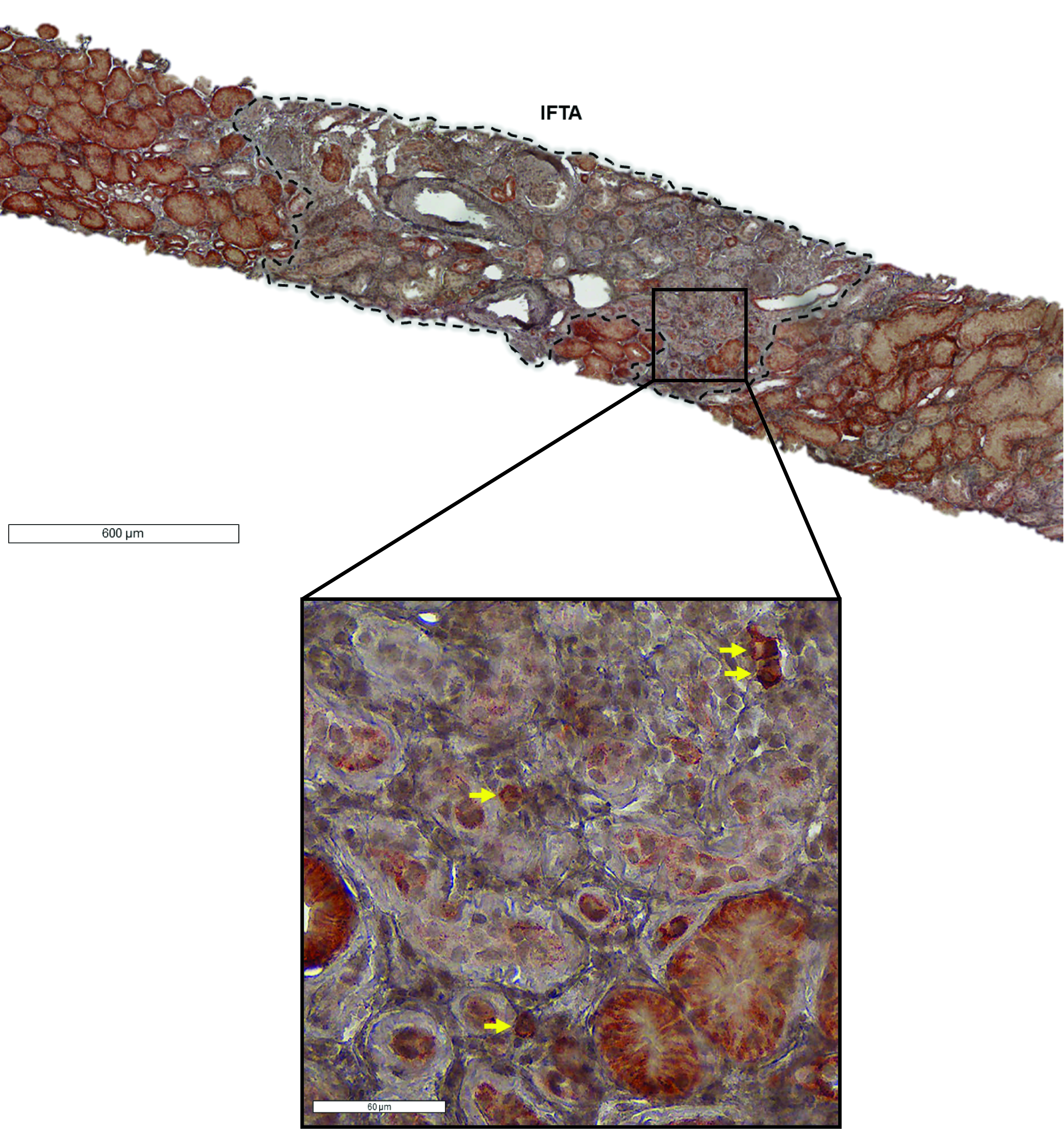

Supplement: online supplemental figure 1 [file lupus-12-2-s001.tif]
